# Commentary: Free Will and Neuroscience: From Explaining Freedom Away to New Ways of Operationalizing and Measuring It

**DOI:** 10.3389/fnhum.2016.00509

**Published:** 2016-10-19

**Authors:** Alvaro M. Dias

**Affiliations:** Clinical Neuroscience Lab “LinC”, Department of Psychiatry, Federal University of São PauloSão Paulo, Brazil

**Keywords:** consciousness, mental states, brain, phenomenological psychology, cognitive science

The long-standing debate on the nature of consciousness and the extent to which free will remains a valid concept has evolved a lot since the publication of Libet's seminal work on neural antecedents of mental states and behavior (Libet et al., 1983). It is very clear now that it is just a matter of time for new sensors, experimental paradigms, and advanced techniques in data analyses provide us with such a high rate of success in forecasting motor and cognitive decisions before they appear to oneself as intentions that the whole discussion about the role of phenomenal consciousness (P-consciousness, following Block's terminology, 1995—or simply P-states) in initiating decision-making and efferent commands will simply disappear (Block, 1995).

At this point, cognitive science will be finally beyond some of its oldest metaphysical debates, while brain studies will reach unprecedented popularity across the humanities, in accordance with the definitive assimilation of the principle that all mental states supervene from neural states, which—should be noted—is not the same as saying that “to each mental construct there is a neurological state,” but rather consistent with the premises that “for each mental construct there is and there was a neurological state” and that “the information processes that culminate in P-states are structured over time in the brain, following bio-computational rules.”

One of the main challenge of the “post-premises” phase is to define the most consistent ways to describe the initiators of the neural signature of P-states and, when applied, related behaviors. It is a challenge that can be broken into several dimensions, with different levels of approachability under current methods and understandings. Whereas topics like the definition of the most relevant sources of neural activity for different types of P-states are getting more and more accessible, matters like the neural coding of symbolic representations and the principles guiding the interactions between these and the computation of environmental clues (sensorial inputs) in the emergence of intentional states of affairs remain far from our understanding of the brain, as they ultimately rely on the decoding of the “mentalese” that biologically instantiates abstract thinking and default mode mind-wandering (Fodor, 1995).

Another point of attention is that of the evolutionary basis of consciousness. As for the initiator challenge, this one can be broken in at least two assignments of different complexities. One related to the possible roles of such a definitive trait to the human kind and another associated with the application of the decoding of mentalese-type of biological algorithms in other species (perhaps, proto-algorithms) so as map the evolution of endophenotypes that culminate in human consciousness (Cabanac et al., 2009).

It is interesting to note that at the same time that the neural branch of cognitive science leaves less space to a metaphysical treatment of mental affairs, its evolutionary branch allows increasingly less room to attempts to reduce defining characters of a species into inactive byproducts of something else. As one would say: after the near neutrality/molecular conceptual revolution that took place in evolutionary biology (Kimura, 1984), we are starting the be able to remap the scope of Neo-Darwinism (Nei, 2005) and more than ever it is clear that defining traits tend to be functional, which herein translates directly into increasingly less room to the assumption that consciousness could be merely epiphenomenal. In contrast to that, it is striking to see how often discussions on neural antecedents (especially when related to other people's findings) are dragged to the hypothesis that consciousness may be a whimsical frivolity of nature or, simply put, an “emergent property.”

Andrea Lavazza's approach to the role of intentional mental states (Lavazza, 2016) can be understood as an alternative to such deadlock, with references to free will that basically do not change the fact that what is really at stake is the scope of P-states. In essence, the hypothesis tries to “integrate neuroscientific research on free will by connecting higher-level concepts with their neural correlates through a psychological operationalization in terms of skills and cognitive functions”; in a sense, Lavazza is in line with authors, like Perlovsky, who say that “believing in free will is extremely important for individual survival” (Perlovsky, 2011, p. 4). According to the former's perspective, these P-states would enhance the expected utility of behavior through the selection of the most convenient bio-computational modes for each situation (i.e., overt or covert attention, deductive reasoning)—which is why it can be classified among the hypotheses that try to shed light into its emergency and fixation. This is obviously no direct response to the claims that conscious has no initiating capacity, as one could always say that these path-defying moments where cognitive programs are selected also emerge from neural antecedents and are no more epiphenomenal than all others state of affairs. Actually, it is the same argument that has been put against the idea that consciousness could be saved as a vetoing process, until the point of no return.

Prof. Lavazza is wise not to put his hypothesis in that gun line, while preserving the ability to present operational concepts, like that of a consciousness index. But that is precisely where a totally new type of epiphenomenal conundrum is introduced: if, for example, the role of a P-state is to trigger a phonological loop or an audiovisual sketch pad that will keep valuable information available for further cognitive operations, it is indistinguishable from what has been called central executive system, which is the instance assumed to operationalize these dedicated working memory modules for the past 40 plus years (Baddeley and Hitch, 1974).

Generalizing this idea, one could argue that the concept that Prof. Lavazza found in the basis of his free will index is the same found by Binet, grandfather of current IQ test, who discussed competences as a matter of consciousness and deliberative power before the enthronement of Franz Ferdinand. In that vein, one could plausibly argue that when free will is assumed to be an irredundant property (which is essential to its conceptual legitimacy) that emerges through the lines of the central executive and other computational dynamics that have been deliberately presented without any reference to the classic debate on consciousness, it is forcedly assumed to be merely epiphenomenal, as it adds a variable without generating any distinguishable output except, perhaps, P-states that merely supervene from these cognitive computations.

There are different types of alternatives to this dilemma (e.g., Perlovsky, 2013). One that has a proximity with Lavazza's approach says that a particular type of competence is instantiated because of specific P-states (not as them), which by that means makes them totally irreducible to any traditional dimension of intelligence. In defense of that view, which I endorse, consciousness can be functionally defined as an unparalleled type of bio-computational platform that allows second level information processing based on inferences of first order mental states, producing sophisticated and yet innate competences like meta-cognition and theory of mind (Metcalfe, 2008; Ando et al., 2013). Consciousness provides us with experiential continuity, while some of the most critical cognitive processes for planning and decision-making (which involve mapping future feelings over current experiences) and social coexistence (which involves mapping other's mental states over ours) comprise inferences that are made with that phenomenological texture as a base and could not be possible in a conscious free agent.

The direct consequences of this approach to free will are clear: volition can be considered “free” as long as it is open to post-drive, second order processing by its instantiation in this computational platform characterized by an experiential sense of continuity. This may be seen as an alternative approach that avoids the aforementioned deadlock, while being aligned with the neural antecedent prerogative.

## Author contributions

The author confirms being the sole contributor of this work and approved it for publication.

## Funding

FAPESP 2015/03931-0.

### Conflict of interest statement

The author declares that the research was conducted in the absence of any commercial or financial relationships that could be construed as a potential conflict of interest.
